# Effect of caffeic acid grafted chitosan loaded quercetin lyophilized powder formulation on avian colibacillosis and tissue distribution

**DOI:** 10.3389/fvets.2024.1470781

**Published:** 2024-10-24

**Authors:** Xin Ren, Sikun Yuan, Juan Ren, Leying Ma, Juxiang Liu, Gengnan Wang

**Affiliations:** ^1^College of Veterinary Medicine, Hebei Agricultural University, Baoding, China; ^2^School of Quality and Technical Supervision, Hebei University, Baoding, China; ^3^Baoding Institute for Food and Drug Control, Baoding, China

**Keywords:** quercetin, *Escherichia coli*, mechanism of bacterial antibacterial, intestinal flora, tissue distribution

## Abstract

Quercetin (QR), recognized as a natural antibacterial ingredient, has found widespread application in the poultry industry. This study investigated the bacteriostatic mechanism and evaluated the *in vivo* inhibitory impact of caffeic acid-grafted chitosan self-assembled micelles loaded quercetin (CA-g-CS/QR) on avian *Escherichia coli* (*E. coli*). The findings indicate that the bactericidal mechanism of CA-g-CS/QR exhibits enhanced efficacy compared to QR alone, disrupting bacterial cell walls, disassembling biofilm structures, and impeding essential components necessary for bacterial growth. Following an avian *E. coli* attack in broilers, CA-g-CS/QR demonstrated the capacity to enhance the population of beneficial bacteria while concurrently decreasing harmful bacteria within the intestinal tract. Moreover, within 3 days of oral administration of CA-g-CS/QR, a significant decrease in *Escherichia* spp. count was evident, resulting in the restoration of broilers to a healthy state. CA-g-CS/QR proved to be a significant and more efficacious solution than QR alone for avian *E. coli* disease. Furthermore, CA-g-CS/QR displayed a broader distribution range and higher concentration within the body. Ten metabolites have been identified in the liver for both QR and CA-g-CS/QR. In conclusion, CA-g-CS/QR has demonstrated a notable capacity to enhance *in vitro* and *in vivo* bacterial inhibitory effects, providing foundation for the clinical application of QR in combating avian *E. coli* infections in broilers.

## Graphical abstract

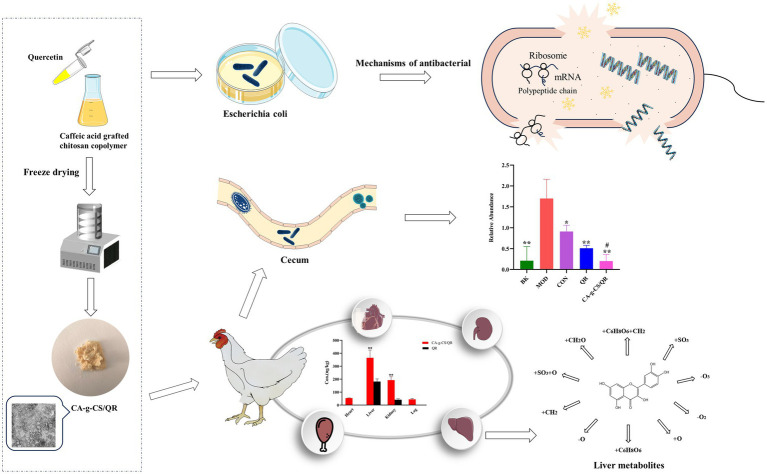

## 1 Introduction

The intestinal tract plays a vital role in both digestion and immune defense, crucial for the growth and development of organisms. Central to its functionality is the intestinal flora, which maintains a harmonious balance capable of modulating the host’s immune system and fortifying defenses against pathogens (1–3). *Escherichia coli* (*E. coli*), a facultative anaerobic microorganism, predominantly affects young animals and birds, thriving in the lower gastrointestinal tract and posing significant threats to health and economic losses in livestock industries (4–7). The escalating trend of antibiotic resistance among *E. coli* strains underscores the urgent need for alternative strategies in livestock farming to ensure food safety and sustainability.

Quercetin (QR), a widely distributed flavonoid compound in plants, has gained attention for its broad antimicrobial properties, effectively combating various pathogens including *E. coli* (8–10). Its mechanisms of action involve disrupting cell walls, interfering with protein synthesis, and exerting metabolic antagonism, making it a promising candidate against multidrug-resistant bacteria (11–14). Studies have demonstrated QR’s ability to reduce pathogenic bacteria in broilers while promoting beneficial microflora, thus enhancing intestinal health and immunity (15).

Chitosan (CS), a natural polymer known for its antimicrobial activity, biodegradability, and biocompatibility, is utilized in food preservation and drug delivery (16–18). Although its efficacy can be limited by solubility issues, chemical modifications and composite strategies enhance its antimicrobial potential against a spectrum of bacteria, including *E. coli* (19, 20). Caffeic acid (CA), a natural fungicide, enhances antimicrobial activity by disrupting bacterial cell structures, which is explored for developing new compounds with therapeutic applications (21–23).

This study investigates caffeic acid-grafted chitosan self-assembled micelles (CA-g-CS) as a carrier for QR, aiming to enhance its solubility and stability under alkaline conditions. Preliminary experiments demonstrate that CA-g-CS/QR improves QR’s bioavailability by 1.67 times compared to the unmodified drug, significantly enhancing its antibacterial efficacy against *E. coli* with reduced MIC and MBC values (24). This research further explores the antibacterial mechanisms of CA-g-CS/QR in white-feathered broilers challenged with avian *E. coli*, assessing its impact on intestinal health and potential clinical applications. These findings lay the groundwork for leveraging QR and CA-g-CS/QR formulations in combating avian *E. coli* infections in broilers, addressing critical concerns in livestock health and food safety.

## 2 Materials and methods

### 2.1 Materials and reagents

Quercetin (≥97%), kaempferol (≥98%), caffeic acid (≥98%), chitosan (deacetylation degree of 90%), the above reagents were purchased from Yuanye Biologicals (Shanghai, China), *Escherichia coli*, chromatography grade acetonitrile was purchased from Merck (Germany), and the kits used in the experiments were purchased from Nanjing Jiancheng Company (China), and the experiments were conducted using ultrapure water prepared for the laboratory.

### 2.2 Experiment animals

The animal experiments were reviewed and approved by the Institutional Animal Ethics Committee of Hebei Agricultural University, with the approval number 2022026, and carried out according to the Guidelines for the Management and Use of Laboratory Animals in China. In this study, 40 healthy 30-day-old white feather broilers were used (Baoding, China). All of the broilers were fed from 1 day of age with adequate basic diet and water, and the basic diet formula met or exceeded the nutritional requirements of the broilers. Before the experiment, broilers were randomly divided into 5 treatment groups.

### 2.3 Preparation of CA-g-CS/QR

Initially, 0.5 g of CS was fully dissolved in a 50 mL solution of 1% acetic acid. Following this, 1.32 g of ascorbic acid and 1 g of CA were introduced. The solution’s pH was meticulously adjusted to 6.0, and a slow nitrogen flow was maintained in the reactor for 60 min. Subsequently, a 10 mol/L hydrogen peroxide solution was introduced to initiate the reaction. The reaction transpired under a continuous nitrogen flow for a duration of 16 h. The resultant solution underwent dialysis in ultrapure water for 72 h, followed by dehydration in a freeze dryer (25).

A CA-g-CS solution with a specific concentration was prepared following the aforementioned procedures. A specific concentration of CA-g-CS solution was prepared according to the above procedure. At the same time, QR was dissolved in methanol and the QR solution was gradually added to the CA-g-CS solution at room temperature with vigorous stirring (1 mg QR per 100 μL methanol solution). The mixture was stirred overnight to promote complete evaporation of methanol. The mixture was stirred overnight to facilitate complete evaporation of methanol.

The CA-g-CS/QR solution was successfully synthesized in our laboratory, and extensive experiments have substantiated the enhanced formulation properties conferred by CA-g-CS on QR (24). The CA-g-CS/QR solution, once prepared, underwent a freeze-drying process to yield lyophilized powder. Subsequently, the resulting powder was subjected to observation under a scanning electron microscope. Morphological features were meticulously examined and compared with those of CA-g-CS and QR, aiming to ascertain the success of the formulation preparation.

### 2.4 Antibacterial mechanism

#### 2.4.1 Determination of anbacterial curve

The antibacterial curve of avian *E. coli* was determined by UV spectrophotometry. The inoculum of 5% (v/v) was inoculated in liquid nutrient broth medium; the same concentration of QR, CA-g-CS/QR, CA-g-CS solution was added as the experimental group, and sterile water was used as the control group. Three parallels of each group were set up, incubated in a shaking bed at 37°C, and the samples were taken at intervals of 2 h. The absorbance at 600 nm was measured continuously for 24 h. The bacteriostatic curve of avian *E. coli* was plotted with the incubation time as the horizontal coordinate and the measured absorbance value as the vertical coordinate (26, 27).

#### 2.4.2 Determination of alkaline phosphatase content

QR, CA-g-CS/QR, CA-g-CS solution was added to the bacterial suspension, the final concentration of the drug solution was 9 mg/mL, and sterile water was used instead of the drug solution as the control group, the samples were taken at regular intervals of 2 h. After centrifugation at 4,000 r/min for 10 min, the supernatant was taken to determine its content by alkaline phosphatase (AKP) kit method, and the change of AKP was detected with the extension of time. Each experimental group was equipped with three parallels.

#### 2.4.3 Determination of nucleic acid and protein leakage

The bacterial suspension was activated and incubated to logarithmic phase, and QR, CA-g-CS/QR, and CA-g-CS solutions were added to make the final concentration of the drug solution 9 mg/mL, and sterile water was set as the control. At the incubation time of 0, 2, 4, 6, 8, 10, and 12 h, samples were centrifuged and the supernatant was taken. The absorbance values were measured at 260 nm and 280 nm with a UV spectrophotometer. This value was used to reflect the content of nucleic acid substances and protein substances in the supernatant, with three parallel experiments in each group.

#### 2.4.4 Crystalline violet staining test

The bacterial suspension was prepared and the experimental group (same concentration of QR, CA-g-CS/QR, CA-g-CS solution) and control group (sterile water) were set up, and the volume of bacterial solution was 1:1 with the volume of drug solution, which was cultured at 37°C in a constant temperature shaker. The suspension was incubated for 24 h, centrifuged at low temperature for 10 min at 6,000 r/min, washed three times with PBS, mixed with crystal violet solution, incubated for 15 min at room temperature and protected from light, and centrifuged to take the supernatant, and the OD590 value was determined. Each group of experiments was equipped with three parallels.

#### 2.4.5 Determination of ATP

The bacterial suspension which was cultured to logarithmic phase after activation was added with QR, CA-g-CS/QR, CA-g-CS solution, so that the final concentration of the drug solution was 9 mg/mL, and incubated in shaker at 37°C for 4 h. The bacterial suspension was centrifuged at 6,000 r/min for 10 min, washed three times with PBS and resuspended, and the resuspended bacterial body was broken by low temperature ultrasonication, and then centrifuged at 8,000 r/min for 10 min, and the cell residues were discarded, and the supernatants were taken to determine the ATP content with an ATP content test kit with 3 parallels in each group.

#### 2.4.6 Determination of half antibacterial concentration (EC50)

The prepared bacterial suspension was inoculated into the liquid medium at an inoculum of 5%, and then different concentrations of QR, CA-g-CS/QR and CA-g-CS were added to the liquid medium, so that the final concentrations of each of them were 0, 2, 4, 9, 18, 36, and 72 mg/mL, respectively, and then the bacteria were cultured at a constant temperature of 37°C for 24 h. The absorbance values were measured at 600 nm by UV spectrophotometer, and the antibacterial rates were calculated. After 24 h, the absorbance values were measured at 600 nm by UV spectrophotometer and the antibacterial rates were calculated. The bacterial growth antibacterial rate was converted into the antibacterial probability value and used as the vertical coordinate, and the concentration of the drug solution was converted into the logarithmic value and used as the horizontal coordinate. Based on the linear regression relationship between the bacterial growth antibacterial probability value (y) and the logarithmic value of the concentration of the drug solution (x), the regression equation of the virulence was solved, and the EC50 was calculated.

$\mathrm{Antibacterial rate}\;\left(\%\right)=\left({OD}_{\mathrm{control}}–{OD}_{\mathrm{treatment}}\right)/{OD}_{\mathrm{control}}\times 100\%$

Note: OD _control_ refers to the absorbance value at 600 nm of the sterile water treatment group, and OD _treatment_ indicates the absorbance value at 600 nm of the experimental group with the addition of different bacteriostatic substances of QR, CA-g-CS/QR and CA-g-CS.

### 2.5 *In vivo* antibacterial experiment

#### 2.5.1 Experimental design

Forty healthy white-feathered broilers with similar body mass at 30 days of age were selected and randomly divided into five groups with eight replicates in each group, namely, the blank group (BK group), the control group (CON group), the model group (MOD group), the QR group and the CA-g-CS/QR group. Except for the BK group, the other four groups were treated with 1.0 × 10^8^ cfu/mL *E. coli* of chicken origin at 1 mL/broiler to establish the morbidity model, and the morbidity of the broilers was observed to confirm the success of modeling. The QR group and the CA-g-CS/QR group received the corresponding solution orally twice daily for three consecutive days at a dose of 18 mg/kg, while the control group was left untreated and the test period was the same as that of the experimental group.

#### 2.5.2 Sample collection

At the end of the experiment, the white-feathered broilers were dissected. The cecum was ligated with a pre-sterilized fine thread, cut off, and the contents of the cecum were taken in a sterile 5 mL centrifuge tube in a biological ultra-clean bench and stored in a refrigerator at −80°C for intestinal microflora analysis.

#### 2.5.3 Analysis of the intestinal microflora

Small fragment libraries were constructed according to the characteristics of the amplified regions, and the libraries were subjected to double-end sequencing based on the illumina NovaSeq sequencing platform. After Reads splicing and filtering, OTUs clustering or ASVs noise reduction, the obtained valid data were subsequently subjected to species annotation as well as abundance analysis to reveal the species composition of the samples; and further *α*-diversity and *β*-diversity analyses could be used to uncover the differences in the community structure among the samples. Up-sequencing was performed using NovaSeq 6000 (Illumina, San Diego, United States).

### 2.6 Tissue distribution

#### 2.6.1 Method creation

Waters TQD LC–MS (Waters, United States). Chromatographic conditions: Waters Acquity UPLC Shield RP18 column (100× 2.1 mm, 1.7 μm, Waters). The mobile phase was acidified acetonitrile-0.2% formic acid in water (40:60, v/v) at a flow rate of 0.3 mL/min.

Mass spectrometry conditions: Ion source: ESI; Detection mode: positive ion mode; Signal acquisition: multiple reaction detection (MRM); Cone hole voltage: 45 V; Collision energy: 25 V; Ion pairs: m/z303 → 229.1 and m/z287 → 164.9 (internal standard).

#### 2.6.2 Preparation of calibration standards

The standard stock solutions of QR and kaempferol (KF) were prepared with acidified acetonitrile/0.2% formic acid (2:3, v/v). A series of working standard solutions of QR at different concentrations (100.0–10000.0 ng/mL) were prepared by diluting the stock solution. A calibration standard solution was made by mixing 200 μL of blank tissue homogenate with 20 μL of the appropriate QR working solution, which was then used to make a series of standard solutions at concentrations of 10.0, 20.0, 50.0, 100.0, 200.0, 500.0 ng/mL, and 1,000 ng/mL.

#### 2.6.3 Tissue sample processing

Take 500 mg of each tissue sample, add 500 μL of saline and mix well for homogenization, centrifuge at 12,000 r/min for 10 min. Take 200 μL aliquot of tissue homogenate, add 20 μL of 40 μg/mL of KF internal standard, 100 μL of 25% HCL and 2 mL of ethyl acetate in sequence, vortex for 2 min. The mixture was centrifuged at 8,000 rpm for 5 min and the supernatant was transferred to a centrifuge tube and concentrated to dryness under nitrogen. The analytes were dissolved in 200 μL of acidified acetonitrile/0.2% formic acid in water (2:3, v/v).

#### 2.6.4 Method validation

A series of QR standard solutions with different concentrations were prepared by dilution. 200 μL of blank tissue homogenate was taken and 20 μL of QR standard solution was added, and treated according to the method under Section 2.6.3. The regression equation of the standard curve of each tissue homogenate was established with the concentration of the substance to be measured in each tissue as the horizontal coordinate and the ratio of the peak area of the substance to be measured to that of the internal standard as the vertical coordinate.

Aspirate 200 μL of each tissue blank homogenate, prepare low, medium and high concentration quality control samples, and analyze the samples according to the method under Section 2.6.3 to obtain the peak area of each component A1. Take another 200 μL of blank tissue homogenate, and process it according to the method under Section 2.6.3, and the residue will be re-solubilized with the standard solutions of low, medium and high concentration, and analyzed by the samples to obtain the peak areas of each component A2. The low, medium and high standard solutions were injected and analyzed to obtain the peak area A3 of each component. Six copies of each sample were prepared in parallel. The ratio of A1–A3 was calculated as the extraction recovery, and the ratio of A2–A3 was calculated as the matrix effect.

Precision and accuracy were assessed by using standards (*n* = 6) at low, medium, and high level concentrations for each organization. Intraday precision and accuracy were calculated based on the results of samples analyzed on the same day; interday precision and accuracy were calculated for three consecutive days of sampling.

#### 2.6.5 Organizational distribution studies

Heart, liver, spleen, lung, kidney, leg and breast tissues of 6 broilers each from QR group and CA-g-CS/QR group *in vivo* antimicrobial experiments were selected, placed in saline, washed, blotted on filter paper, and divided. Store in the refrigerator at −80°C for use.

### 2.7 Identification of QR metabolites *in vivo*

#### 2.7.1 Methods of detection

Q EXACTIVE PLUS UPLC-MS (Thermo Scientific). Chromatographic conditions: Vanquish Flex UPLC, HSS T3 column (2.1*100 mm, 1.8 μm), mobile phase 0.1% formic acid in water (A)-acetonitrile (B), gradient elution, 0 ~ 0.5 min, 98%A; 0.5 ~ 15 min, 98% ~ 60%A; 15 ~ 18 min, 60% ~ 10%A; 18 ~ 20 min, 10%A; 20 ~ 21 min, 10% ~ 98%A; 21 ~ 24 min, 98%A; flow rate 0.3 mL/min, column temperature 40°C.

Mass Spectrometry Conditions: Spray Voltage: −3.2 kV/+3.8 kV, Ion Transfer Tube Temperature: 325°C, Atomization Temperature: 350°C, Scanning Mode: fullmass-ddMS2, Scanning Range: 100–1,000 for first stage, auto for second stage.

Compound Discoverer software was used to retrieve possible metabolites through its own metabolic pathway.

#### 2.7.2 Metabolite detection

Two groups of white-feathered broilers were dosed with QR and CA-g-CS/QR respectively, and there was a blank group at the same time. After 2 h, the livers were taken and placed in saline, washed, blotted on filter paper, and divided. The samples were processed and tested according to the method in 2.6.3.

## 3 Results

### 3.1 Scanning electron microscopy morphological observations

**Figure 1:**
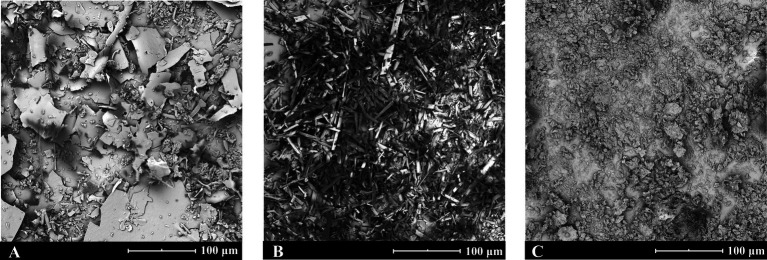
CA-g-CS, QR and CA-g-CS/QR scanning electron microscope view. **(A)** Scanning electron microscope view of solid CA-g-CS with a particle size of about 46.9 ± 6.2 μm; **(B)** Scanning electron microscope view of QR with a particle size of about 37.3 ± 4.9 μm; **(C)** Scanning electron microscope view of solid CA-g-CS/QR with a particle size of about 12.1 ± 3.6 μm.

The scanning electron microscopy results, as illustrated in Figure 1: (A) solid CA-g-CS exhibits a lamellar structure with an approximate diameter of 46.9 ± 6.2 μm; (B) QR assumes a rod-like crystalline state with a diameter of about 37.3 ± 4.9 μm; (C) the lyophilized powder of CA-g-CS/QR presents a smaller flocculent structure, featuring a diameter of around 12.1 ± 3.6 μm.

### 3.2 Antibacterial curves

**Figure 2:**
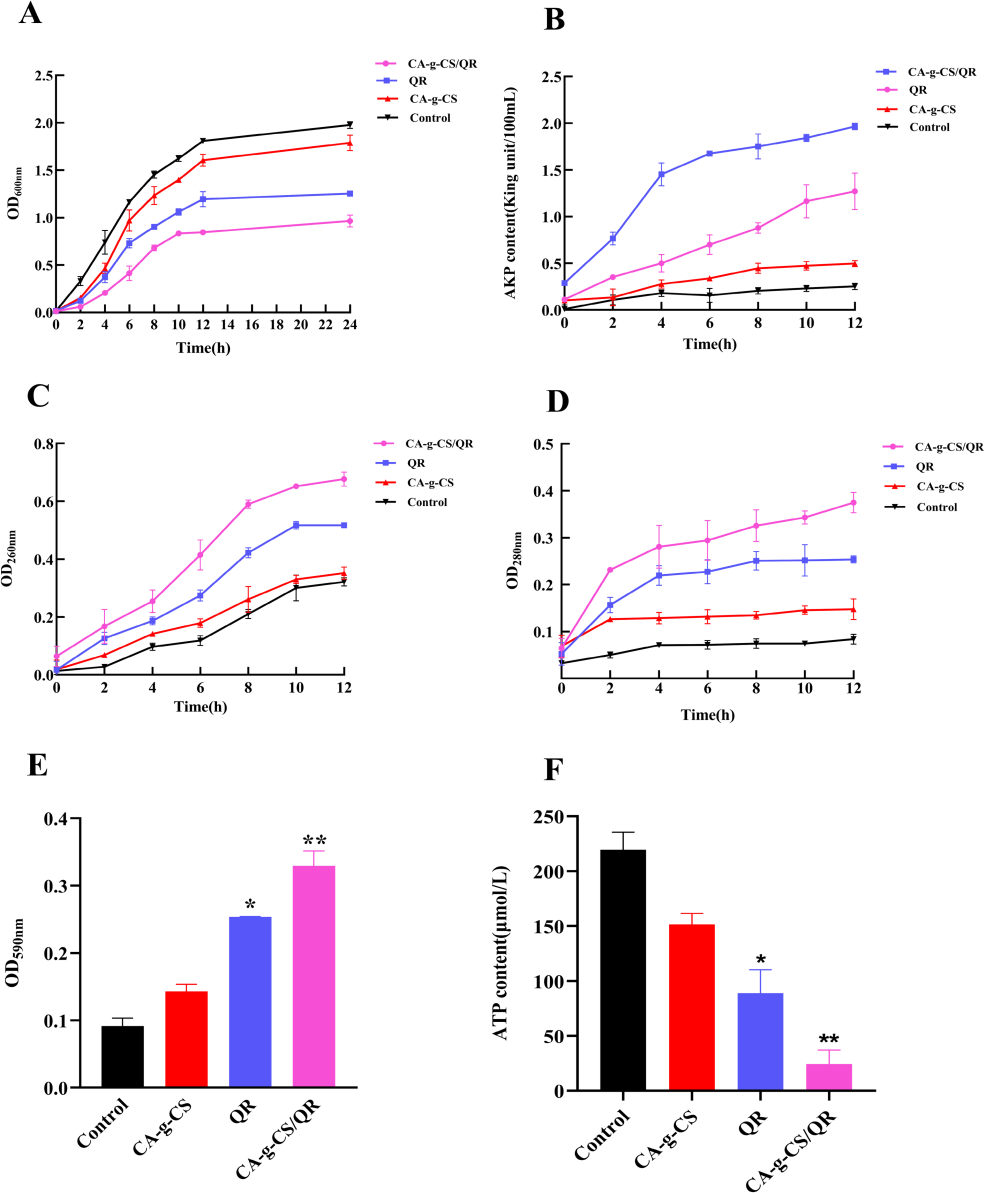
Results of each index of antibacterial mechanism. **(A)** Antibacterial curves of QR, CA-g-CS/QR, CA-g-CS, and control. **(B)** Experimental results of AKP content measurement. **(C)** Experimental results of nucleic acid leakage content measurement. **(D)** Experimental results of protein leakage content measurement. **(E)** Crystalline violet staining results (*n* = 3, *p < 0.05, **p < 0.01). **(F)** Experimental results of ATP content determination (*n* = 3, *p < 0.05, **p < 0.01).

Figure 2A illustrates the results indicating robust bacterial growth in the control group. Compared to this, both the QR and CA-g-CS groups exhibited reduced OD values, signifying diminished bacterial growth. Remarkably, the OD value change in the CA-g-CS/QR group was minimal.

### 3.3 Determination of AKP content

As depicted in Figure 2B, the consistently lower AKP activity in the control group suggests superior growth of pathogenic bacteria with an intact cell wall structure. Both CA-g-CS and QR, as well as the CA-g-CS/QR combination, proved effective in disrupting the integrity of the bacterial cell wall. Notably, the impact of CA-g-CS/QR surpassed that of QR alone, affirming the enhanced effectiveness of the combined formulation. This substantiates the notion that CA-g-CS can augment the bacteriostatic capabilities of QR.

### 3.4 Determination of nucleic acid and protein leakage levels

In Figure 2C, the control group showed a progressive rise in nucleic acid leakage due to regular bacterial growth and apoptosis over the incubation period. Conversely, the absorbance value at 260 nm notably surged in the CA-g-CS/QR group, indicating considerable cellular content leakage. These observations suggest a probable occurrence of irreversible damage to the cytoplasmic membrane, potentially leading to the loss of essential cellular components such as DNA and RNA, ultimately culminating in cell death.

As depicted in Figure 2D, over the incubation period, the control group exhibited increased protein content attributed to bacterial growth and concentration elevation. Both the QR and CA-g-CS groups experienced content spillage due to bacterial disruption, whereas the CA-g-CS/QR group displayed a notably swifter decrease in protein content.

### 3.5 Analysis of crystalline violet staining results

In this study, crystal violet staining served to assess bacterial cell membrane damage, as illustrated in Figure 2E. CA-g-CS, CA-g-CS/QR, and QR exhibited varying degrees of impact on biofilm integrity. Notably, CA-g-CS/QR induced more severe damage to the biofilm, suggesting that CA-g-CS contributes to enhancing the bacterial inhibitory capability of QR.

### 3.6 Determination of ATP content

The impact of CA-g-CS, CA-g-CS/QR, and QR on the ATP content of avian *E. coli* (Figure 2F) suggests an influence on bacterial ATP synthesis or consumption. Our results suggest that the modification with CA-g-CS enhances QR’s capacity to impair the bacterial cell membrane, consequently reducing bacterial ATP content.

**Figure 3:**
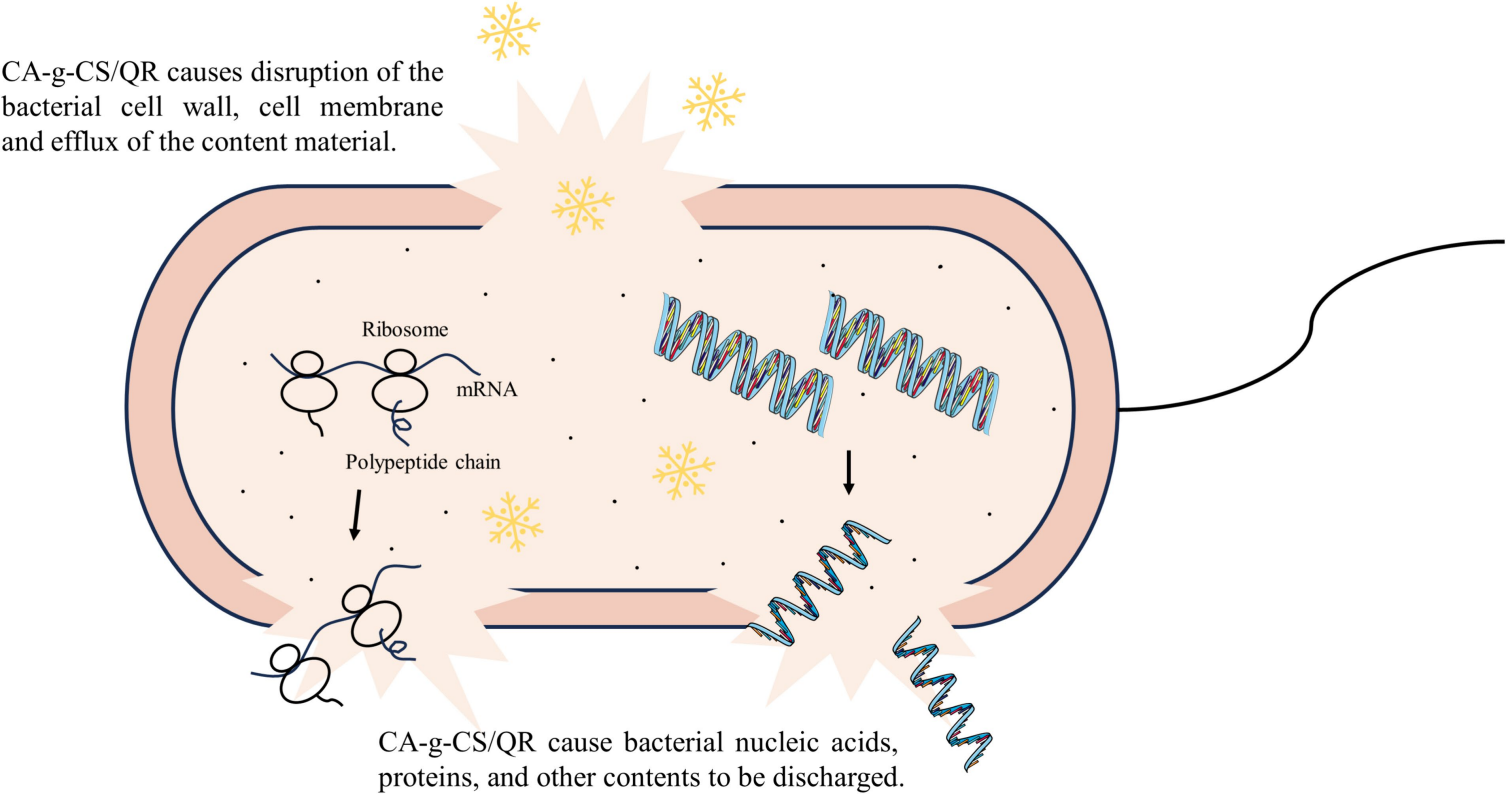
Diagram of CA-g-CS/QR antibacterial mechanism. CA-g-CS/QR destroys bacterial cell walls and cell membranes and causes the efflux of nucleic acids, proteins and other contents.

Based on multiple inhibition mechanism experiments outlined above, it is evident that CA-g-CS/QR disrupts the bacterial cell wall and membrane, resulting in substantial content leakage and ultimately culminating in bacterial death through lysis. This antibacterial mechanism of CA-g-CS/QR is depicted in Figure 3.

### 3.7 EC50 measurement

The calculated CA-g-CS, CA-g-CS/QR and QR EC50 were 10.23 mg/mL, 6.76 mg/mL, and 8.71 mg/mL, which showed that CA-g-CS, CA-g-CS/QR and QR inhibited avian *E. coli* to different degrees, but avian *E. coli* was more sensitive to CA-g-CS/QR.

### 3.8 Results of the analysis of the intestinal flora

Throughout the test period, daily observations were made regarding the health status of broilers. Under normal conditions, broilers exhibited columnar spiral conical feces, light gray-green in color, with white urate bands attached to the fecal surface, presenting a soft and fluffy texture. During episodes of diarrhea, the manifestations varied, displaying diluted feces in shades of white, red, or green. Broilers subjected to the modeling of avian *E. coli* infection exhibited signs of depression, huddling, and lying down, accompanied by disheveled feathers. However, among the experimental groups, broilers treated with CA-g-CS/QR demonstrated heightened agility, robust appetite during feeding, assertive food and water intake, maintained smooth feathers, and exhibited increased body weight compared to other groups.

#### 3.8.1 Quality control of raw sequencing data

Through the quality control of the original data, according to the sequencing of the data obtained by computational processing, the original data is 645737.7, the filtered data is 612307.1, the effective data is 518545.6, the average length is 413.0 bp, the average effective rate is 80%, the quality of sequencing data information analysis is reliable.

#### 3.8.2 OTU analysis

**Figure 4:**
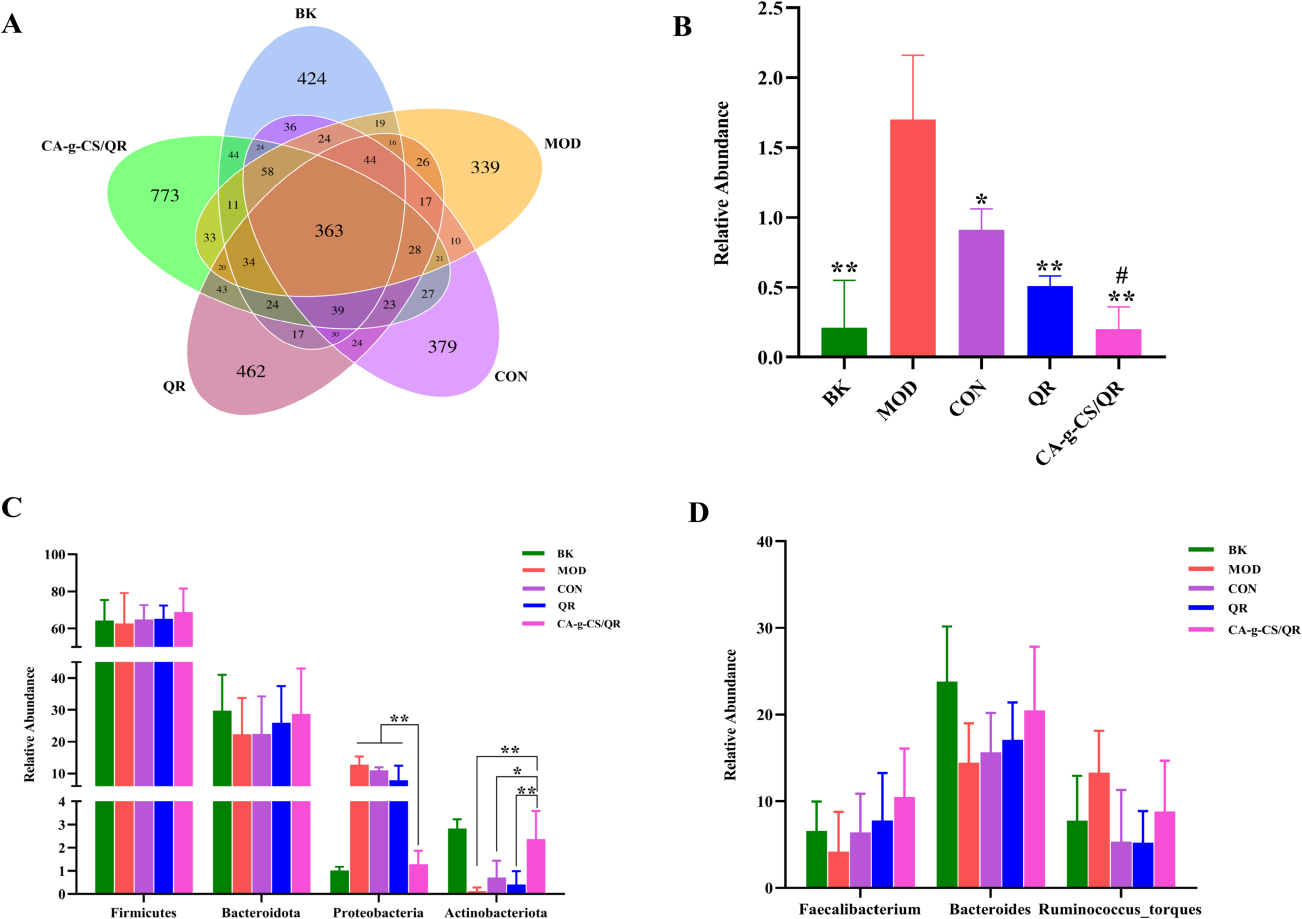
Sample OTUs Venn diagram and relative abundance histogram of flora. **(A)** Sample OTUs Venn diagram. **(B)** Histogram of relative abundance of *Escherichia* spp. (*p < 0.05, **p < 0.01 vs. MOD group. ^#^*p* < 0.05 vs. CON group). **(C)** Relative abundance of dominant flora at the phylum level (*p < 0.05, **p < 0.01). **(D)** Relative abundance of dominant flora at the genus level.

As can be seen from Figure 4A, the BK group contains 1,207 OTUs, the MOD group contains 1,063 OTUs, the CON group contains 1,147 OTUs, the QR group contains 1,210 OTUs, and the CA-g-CS/QR group contains 1,565 OTUs; of which the core OTUs among the three are 363, the unique OTUs of the BK group are 424, the MOD group exclusive OTUs were 339, CON group exclusive OTUs were 379, QR group exclusive OTUs were 462, and CA-g-CS/QR group exclusive OTUs were 773. The test results showed that the number of OTUs decreased in the MOD and CG groups and increased in the QR and CA-g-CS/QR groups compared with that in the BK group, and the number of OTUs increased significantly more in the CA-g-CS/QR group than in the QR group.

#### 3.8.3 Species annotation and analysis

The *Escherichia* spp. changes depicted in Figure 4B revealed a significant increase in *Escherichia* spp. count in the MOD group compared to BK, affirming the success of the modeling process. Both the QR and CA-g-CS/QR groups displayed decreased *Escherichia* spp. counts compared to the CON group, signifying a certain therapeutic effect against avian *E. coli* infections. Notably, the *Escherichia* spp. count in the CA-g-CS/QR group significantly decreased compared to the CON group, reaching levels akin to the normal-fed BK group. This superiority of the CA-g-CS/QR group over the QR group further substantiates the beneficial therapeutic impact of CA-g-CS/QR on broilers’ intestinal tracts infected with avian *E. coli*. This treatment not only enhances the broilers’ resistance against avian *E. coli* infection but also plays a vital biological role in safeguarding the health of the broiler intestinal tract.

Figure 4C illustrates a histogram detailing the distribution of highly abundant phylum-level microorganisms, predominantly composed of Firmicutes, Bacteroidota, Proteobacteria, Actinobacteria, and others, collectively constituting over 90% of the total microorganisms. As depicted in Figure 4C, CA-g-CS/QR positively influenced the distribution of bacterial flora in the intestinal tract compared to the MOD group. Relative to the CON group, CA-g-CS/QR demonstrated enhanced ability to elevate the relative abundance of Firmicutes and, to some extent, Bacteroidota and Actinobacteriota. This increase fostered the growth of beneficial bacteria within the broiler’s intestinal tract. Importantly, CA-g-CS/QR’s capacity to boost the abundance of beneficial bacteria exceeded that of QR alone. Consequently, CA-g-CS/QR significantly improved flora distribution in the broiler intestine following the challenge, enhancing the count of beneficial bacteria and mitigating the proliferation of harmful bacteria. This ultimately contributes to sustaining intestinal health in broilers compared to QR alone.

Figure 4D is a histogram of the distribution of microorganisms of genera with large relative abundance at the genus level, which mainly included: Bacteroides, Ruminalococcus, and Faecalibacterium, occupying more than 90% of the total microorganisms, which belong to the most common bacterial genera in the intestinal tract of broilerss. The relative abundances of Bacteroides, Ruminalococcus and Faecalibacterium in each group were as follows: 23.82, 7.75, and 6.60% in the BK group; 14.46, 13.31, and 4.21% in the MOD group; 15.65, 5.36, and 6.40% in the CON group; and 17.12, 5.24, and 7.79% in the QR group.; 20.47, 8.81 and 10.52% in the CA-g-CS/QR group.

#### 3.8.4 Alpha diversity

The greater the chao and shannon indices in *α*-diversity, the higher the diversity, the smaller the simpson index, the higher the diversity; the greater the pielou_e index, the more evenly distributed the community. As shown in Table 1, compared with the CON group, the chao index, shannon index, and pielou_e index were higher in the QR and CA-g-CS/QR groups, indicating that the QR and CA-g-CS/QR groups were better than the CON group in terms of diversity of the community and uniformity of the species distribution, and CA-g-CS/QR group is better than QR group.

**Table 1:** Sample diversity index.

Group name	Chao	Shannon	Simpson	Pielou_e
BK	382.67	6.26	0.94	0.74
MOD	289.63	5.25	0.91	0.65
CON	320.71	5.54	0.96	0.67
QR	350.49	5.52	0.93	0.67
CA-g-CS/QR	361.60	6.21	0.93	0.75

#### 3.8.5 Beta diversity

**Figure 5:**
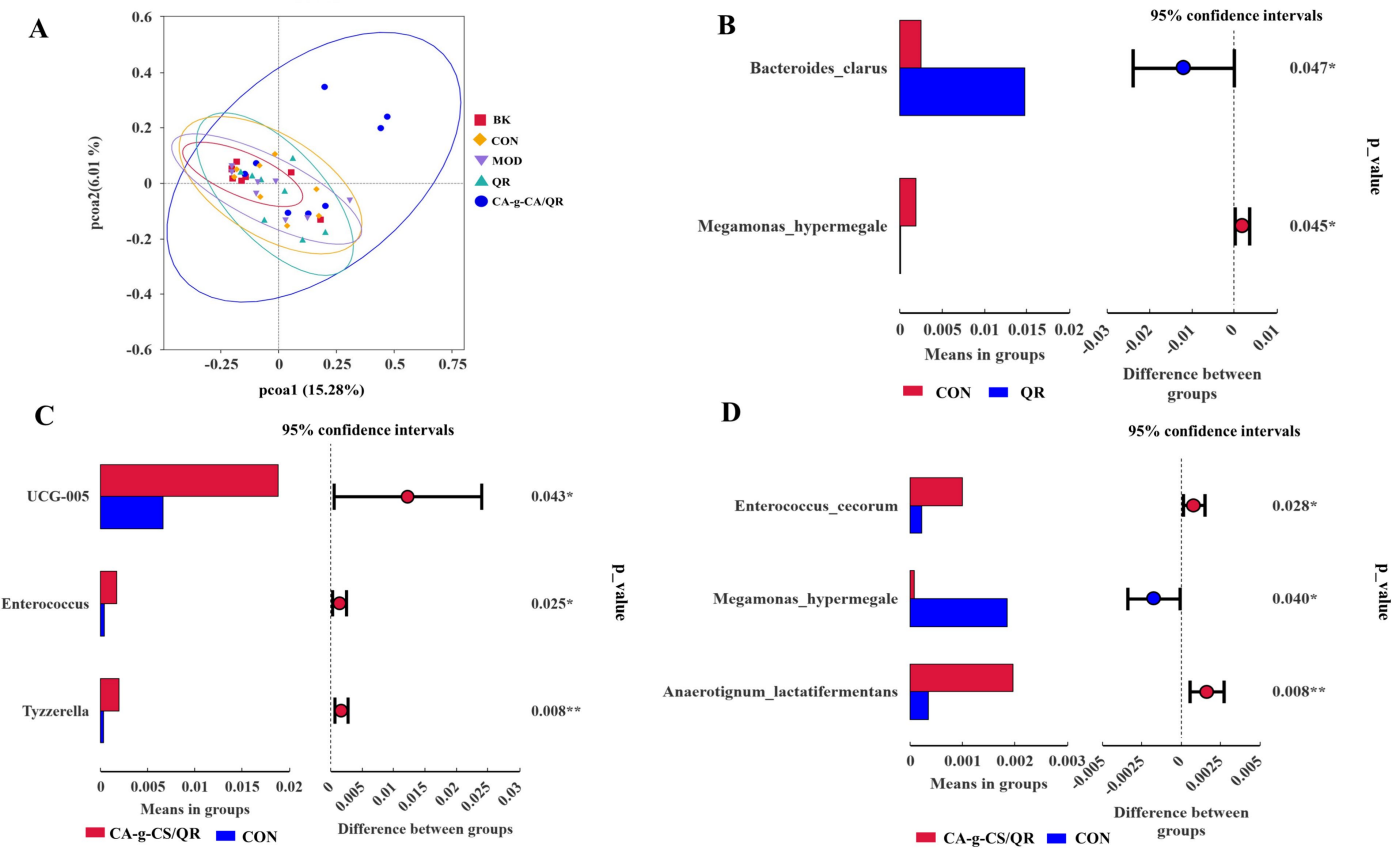
Analysis results of PcoA and T test. **(A)** PcoA results analysis graph. **(B)** Species difference analysis between QR group and CON group T-test group at species level (*p < 0.05). **(C)** Species difference analysis between CA-g-CS/QR group and CON group T-test group at genus level (*p < 0.05, **p < 0.01). **(D)** Species difference analysis between CA-g-CS/QR group and CON group T-test group at species level (*p < 0.05, **p < 0.01).

The relationships between microbial communities in the different treatment groups are represented by PCoA plots (Figure 5A). The microbial communities in each group formed separate clusters of clusters with overlap between groups. In contrast, the microbial community in the CA-g-CS/QR group formed clusters that were clearly separated from the other groups.

#### 3.8.6 T-test analysis

As shown in Figure 5B, *Bacteroides clarus* and *Megamonas hypermegale* were significantly higher in the QR group compared with the CON group (*p* < 0.05). In Figure 5C, at the genus level, UGG-005 and Enterococcus were significantly different in the CA-g-CS/QR group compared with the CON group (*p* < 0.05), and the genus Tyzzerella was highly significantly different between the two groups (*p* < 0.01), whereas there were no species that were significantly different between the QR group and the CON group at this taxonomic level. At the species level (Figure 5D), *Enterococcus cecorum* and *Megamonas hypermegale* were significantly higher in the CA-g-CS/QR group compared with the CON group (*p* < 0.05), and Anaerotignum lactatifermentans anaerobes were highly significant higher than in the control group (*p* < 0.01). It indicates that the increase of beneficial bacteria in the CA-g-CS/QR group reflects the richness of the intestinal flora.

### 3.9 Organizational distribution results

#### 3.9.1 Method specificity

**Figure 6:**
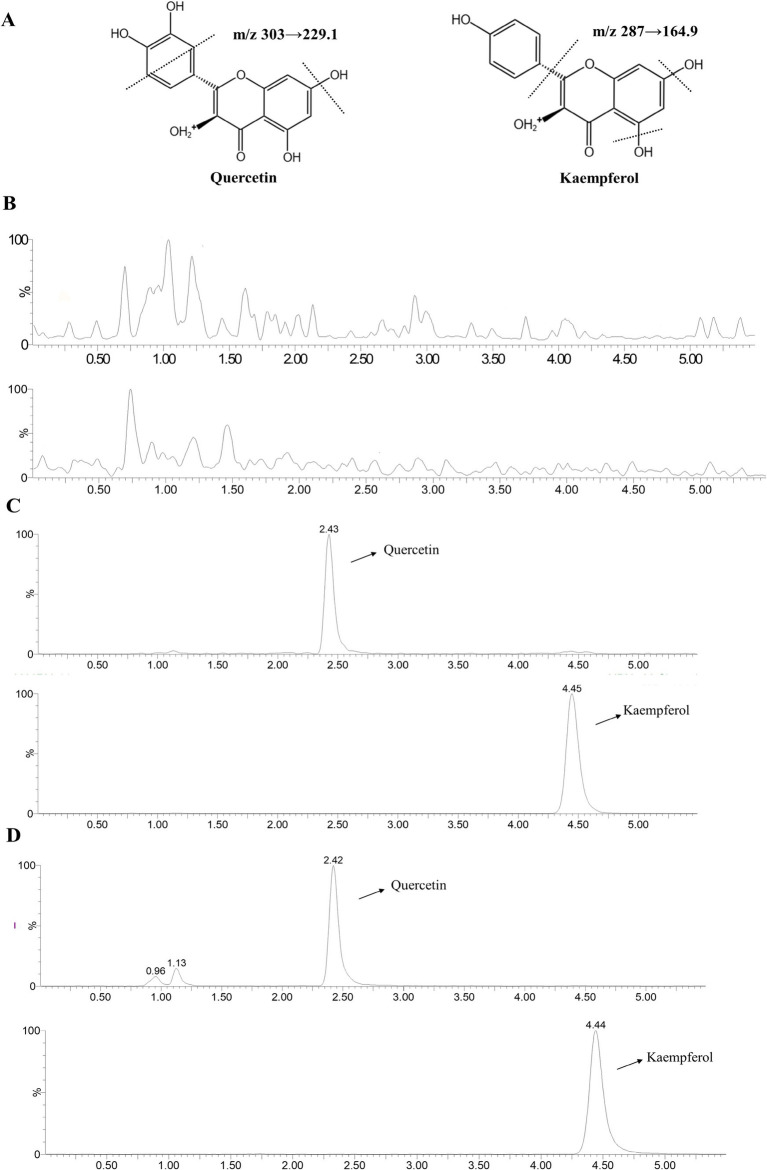
LC–MS chromatogram of QR and KF. **(A)** Chemical structural formulae of QR and KF. **(B)** LC–MS chromatograms of pooled blank tissue. **(C)** Blank tissue with QR at concentration of 500 ng/mL and KF with 20 μL of 40 μg/mL. **(D)** The tissue at 2 h of a broiler following oral administration of QR. QR and KF were detected at m/z 303 → 229.1 and 287 → 164.9, respectively.

The chemical structures of QR and KF are shown in Figure 6A, which also shows representative chromatograms of blank tissue (Figure 6B), blank tissue containing QR and KF standards (Figure 6C), and tissue extract from broilers after oral administration of QR (Figure 6D). Under the assay conditions, no interference peaks were observed in the blank tissues. The retention time was 2.43 min for QR and 4.48 min for KF.

#### 3.9.2 Method validation

Utilizing the measured concentrations of QR and KF within the matrix as the independent variable and the ratio of the peak area of the measured substance to the actual peak area of the internal standard as the dependent variable, linear regression calculations were executed to derive the regression equation, thereby establishing the standard curve. The outcomes are presented in Table 2. Precision, accuracy, and extraction recovery results are detailed in Table 3, demonstrating conformity with testing conditions for biological tissue samples without the interference of matrix effects.

**Table 2:** Linear equations of QR in various tissue samples of white feather broilers.

Tissue	Linear range (ng/mL)	Linear regression equation	*R* ^2^
Heart	5–200	Y = 0.0001x + 0.0028	0.9952
Liver	20–1,000	Y = 0.0003x + 0.0027	0.9991
Kidney	20–500	Y = 0.0002x + 0.0022	0.9944
Leg	5–200	Y = 0.0003x + 0.0061	0.9977

**Table 3:** Precision, accuracy and recovery of the method for the determination of QR in tissues.

	Conc. (ng/mL)	Within-run (*n* = 6)		Between-run (*n* = 6, three runs)		Recovery (%)
		Precision (RSD %)	Accuracy (%)	Precision (RSD %)	Accuracy (%)	
Heart	10	8.18	110.5 ± 15.2	8.84	111.8 ± 16.5	99.8 ± 19.1
	50	10.99	93.5 ± 11.9	5.02	96.7 ± 5.6	87.0 ± 18.6
	200	3.39	100.0 ± 3.5	3.37	99.3 ± 3.5	82.8 ± 11.3
Liver	50	6.46	92.8 ± 6.6	7.29	93.5 ± 7.5	85.8 ± 11.2
	200	4.98	100.7 ± 5.1	5.44	99.7 ± 5.5	82.5 ± 11.9
	500	3.57	97.9 ± 3.5	4.17	97.7 ± 4.1	100.8 ± 9.2
Kidney	50	5.64	98.1 ± 6.9	4.82	98.0 ± 5.9	105.9 ± 2.3
	200	6.76	99.8 ± 7.2	7.63	100.5 ± 8.2	82.4 ± 12.9
	500	4.28	98.8 ± 4.3	5.13	98.2 ± 5.2	104.5 ± 10.1
Leg	10	4.51	94.3 ± 13.3	3.05	95.0 ± 9.0	97.7 ± 3.3
	50	2.96	96.7 ± 4.0	2.27	97.6 ± 3.1	93.1 ± 2.6
	200	3.31	99.8 ± 3.6	3.50	99.7 ± 3.8	99.8 ± 7.2

Each value represents the mean ± SD (*n* = 6).

#### 3.9.3 Application to tissue distribution

**Figure 7:**
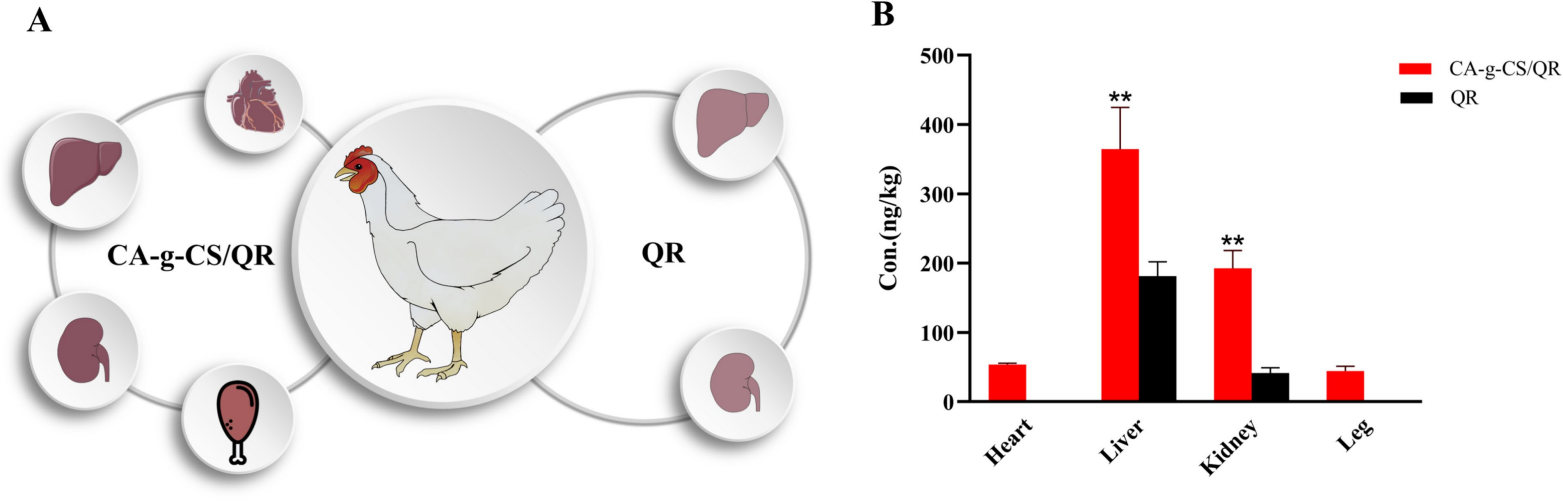
QR and CA-g-CS/QR tissue distribution. **(A)** Tissue distribution modeling of CA-g-CS/QR and QR in white-feathered broilers, dark tissue color means high drug content, light color means less drug content. **(B)** Plot of *in vivo* tissue distribution results in white-feathered broilers after oral of QR or CA-g-CS/QR (*n* = 6, *p < 0.05, **p < 0.01).

The distribution comparison between CA-g-CS/QR and QR in white-feathered broilers is illustrated in Figure 7A, while the specific drug concentration distribution across tissues is detailed in Figure 7B. Notably, the CA-g-CS/QR group exhibited a notably broader distribution range than the QR group. Distribution was observed in the heart, liver, kidney, and leg of the CA-g-CS/QR group, with a higher distribution noted in liver and kidney tissues at equivalent doses. This suggests a potential prolongation of QR elimination facilitated by CA-g-CS. Conversely, QR was solely detected in the liver and kidney in the QR group. Both administration groups predominantly concentrated in tissues with abundant blood flow, namely the liver and kidney, with minimal content found in the heart and legs. This observation aligns with the notion that QR metabolism primarily occurs in the liver and its subsequent excretion via the kidneys. CA-g-CS/QR expanded QR distribution within heart and leg tissues, prolonging QR’s relative retention time in the liver and kidney tissues. This adaptation is presumed to enhance QR’s bioavailability *in vivo*.

### 3.10 Analysis of QR metabolites *in vivo*

**Figure 8:**
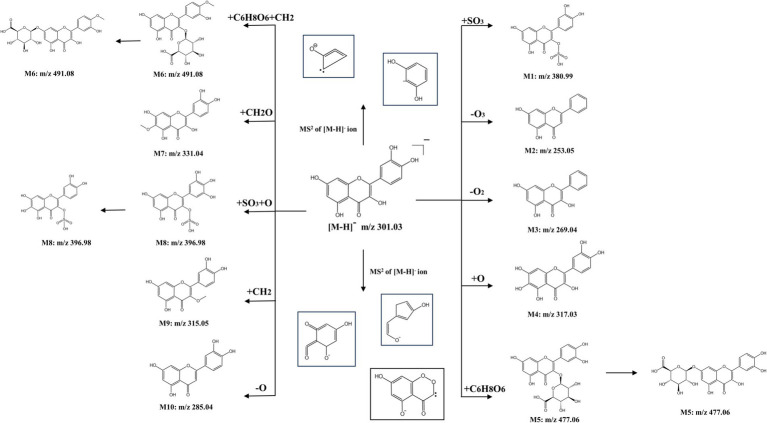
Predicted structural formulae for QR and CA-g-CS/QR metabolites. The same numbered metabolites being isomers.

Comprehensive analysis of liver extracts from the QR and CA-g-CS/QR groups, compared with the blank control group, revealed the presence of 10 suspected QR metabolites. Notably, three metabolites displayed dual peaks within the same ion chromatogram, indicating the possibility of these compounds being isomers. Among these, as depicted in Figure 8, M1, M5, M6, M7, and M8 corresponded to those previously identified in animal tissues, organs, or human blood and urine, as reported in existing literature. Additionally, no metabolites exceeding m/z > 500 were detected in tissues, organs, blood, or urine, aligning with existing literature findings (28–30).

QR sulfate is an important metabolite of QR. m/z of M1 measured in the sample was 380.99, which means 80 Da more than the molecular ion peak of QR that is one sulfated molecular weight, and the fitted elemental composition was C_15_H_9_O_10_S, therefore, it was speculated that M1 might be QR monosulfate (QR + SO_3_).

M2 measured m/z is 253.05, 48 Da that is three oxygen molecular weights less than the molecular ion peak of QR, and the fitted elemental composition is C_15_H_9_O_4_, which is presumed to be the metabolite of QR de-trioxidation. M3 measured m/z is 269.04, 32 Da which is two oxygen molecular weights less than the molecular ion peak of QR, and the fitted elemental composition is C_15_H_9_O_5_, which is presumed to be the metabolite of QR de-dioxidation. The measured m/z of M4 is 317.03, which is one oxygen molecular weight more than the molecular ion peak of QR, and the fitted elemental composition is C_15_H_10_O_8_, which is presumed to be the metabolite of QR + O.

QR glucuronide is another important metabolite of QR, and the m/z measured for M5 was 477.06, which may be QR glucuronide (QR + C_6_H_8_O_6_), with a possible substitution position of either the 3-position or the 7-position, and thus possibly QR-3-O-glucuronide or QR-7-O-glucuronide.

The measured m/z of M6 was 491.08, and its structure was presumed to be methyl QR glucuronide, which may be substituted at 3 or 7 positions, and thus may be methyl QR glucuronide with different substitution positions.

M7 m/z is 396.98, and the fitted elemental composition is C_15_H_9_O_11_S, which is presumed to have a possible structure of QR + SO_3_ + O, and there may be substitutions at different positions. M8 has a measured m/z of 331.04, and the fitted elemental composition is C_16_H_12_O_8_, and therefore it is presumed to have a possible structure of QR + CH_2_O. The m/z of M9 is 285.04, which is one oxygen molecular weight less than the molecular ion peak of QR, and the presumed elemental composition is C_15_H_10_O_6_, which means that M9 may be a QR deoxygenation metabolite. m/z measured for M10 was 315.05 and the fitted elemental composition was C_16_H_11_O_7_, which is presumed to be a QR methylation product.

## 4 Discussion

Scanning electron microscopy revealed that the prepared CA-g-CS/QR was uniformly dispersed without aggregation, and its particle size was smaller compared to the original drug, which presented as larger rod-like crystals. The differences in morphology and particle size between the lyophilized CA-g-CS/QR solution and its individual components (CA-g-CS and QR) clearly demonstrate the successful preparation of the CA-g-CS/QR lyophilized powder.

Bacterial concentration in the liquid can be assessed using absorbance at 600 nm; higher absorbance values indicate higher bacterial concentrations. By examining the antibacterial curves of CA-g-CS, CA-g-CS/QR, and QR, their inhibitory effects against avian *E. coli* were evaluated. Notably, CA-g-CS/QR exhibited a significantly stronger antibacterial effect against avian *E. coli*. Alkaline phosphatase (AKP), an enzyme located between the bacterial cell wall and membrane, is not detectable outside the cell under normal growth conditions. However, when the cell wall is disrupted, AKP leaks into the extracellular space, catalyzing the hydrolysis of disodium benzene phosphate to produce free phenol and phosphoric acid. The resulting phenol, in an alkaline solution, reacts with 4-aminoantipyrine and is oxidized by potassium ferricyanide to produce a red quinone derivative, which serves as an indicator of enzyme activity (31). CA-g-CS/QR had a more pronounced effect than QR alone, supporting the notion that CA-g-CS enhances the bacteriostatic properties of QR.

Cell membrane integrity is crucial for cellular protection. When disrupted, small molecules are initially released, followed by larger molecules such as DNA and RNA (32). The release of intracellular substances is a reliable indicator of membrane integrity. Nucleic acids, which are essential for protein synthesis, play a critical role in cellular functions; loss of these can lead to cell death (33). Proteins, as fundamental components of life and cellular activity (34), also serve as markers of membrane disruption. The changes observed in bacterial cells, including cell membrane cleavage and altered permeability, likely result from QR-induced membrane damage, leading to cell lysis and content leakage. CA-g-CS amplifies QR’s effects, leading to significant protein loss.

Crystal violet, a hydrophobic dye, is inhibited by an intact bacterial cell membrane, preventing its entry. However, when the membrane is compromised, the dye penetrates and stains the membrane (35). The cell membrane functions as a natural protective barrier for bacteria, contributing to their resistance to environmental challenges and recurrent infections. Biofilm formation, a key factor in antibiotic resistance (36), was notably suppressed by CA-g-CS/QR in avian *E. coli*, suggesting its potential as a treatment to combat drug-resistant strains.

Adenosine triphosphate (ATP), a critical energy carrier in living organisms, directly affects cellular metabolism. Alterations in ATP content profoundly impact cellular physiological and pathological processes (37). Declining ATP levels are typically associated with apoptosis, necrosis, or cellular toxicity (38, 39). Our findings confirmed that QR negatively affects bacterial cell membranes, leading to a significant reduction in ATP levels.

Microbial communities in the chicken intestine, including Firmicutes, Bacteroidota, Proteobacteria, and Actinobacteria, are essential for maintaining a balanced intestinal microbiota, which is vital for host development, immunity, and metabolism (40). Firmicutes, dominant in the intestinal flora, produce resilient endospores that ensure survival and transmission upon germination. Bacteroidota, widely distributed in the intestinal flora, contribute to carbohydrate degradation (41–43), while Actinobacteriota support animal growth and defense. Key genera such as Bacteroides, Ruminalococcus, and Faecalibacterium play important roles in nutrient digestion and absorption (44). CA-g-CS/QR was shown to regulate microorganisms in the cecum by increasing the abundance of beneficial genera, thereby promoting intestinal health and supporting the growth of broilers.

QR’s clinical utilization is limited by its low oral bioavailability due to poor water solubility and absorption. Our preliminary studies showed that CA-g-CS/QR significantly enhances QR’s bioavailability *in vivo*, likely due to increased stability and reduced clearance in the body. This prolonged tissue retention time of QR delays its elimination. These findings provide important insights for the further development and clinical application of QR. In both the QR and CA-g-CS/QR groups, 10 metabolites were identified, indicating extensive metabolic transformation of QR in the liver, primarily through sulfation. Tissue distribution studies revealed that QR is highly concentrated in the liver with extended retention time, which likely accounts for its extensive liver metabolism. Further investigation is required to determine the impact of these metabolites on QR’s bioactivity and bioavailability.

## 5 Conclusion

Conclusively, this study unveils QR’s inhibitory mechanism on avian *E. coli* through a comprehensive assessment encompassing growth curve analysis, crystal violet staining, nucleic acid and protein leakage, AKP, and ATP content. CA-g-CS significantly augments QR’s antibacterial efficacy, potentially owing to the combined influence of chitosan and caffeic acid. *In vivo* assessments showcase CA-g-CS/QR’s efficacy in ameliorating post-*Escherichia coli* infection conditions in broilers. It facilitates an increase in beneficial bacterial presence, reduces harmful bacteria, and more effectively modulates community diversity with uniform species distribution compared to QR alone. Moreover, scrutinizing drug tissue distribution unveils prolonged and intensified presence of QR’s active ingredients, bolstering pharmacodynamic effects. Notably, the liver predominantly metabolizes orally administered QR, contributing to its diminished bioavailability due to the initial pass effect.

This study provides foundational data supporting the development and clinical application of QR, suggesting a potential strategy to enhance its therapeutic efficacy by prolonging its elimination rate. This approach holds promise for addressing the challenge of drug resistance in livestock and poultry. The CA-g-CS synthesized in this experiment demonstrates favorable physical and chemical properties, obviating the need for additional carriers typically required for traditional micelles. It shows potential for encapsulating other drugs to improve clinical efficacy. Future research directions could explore the encapsulation of various drugs by CA-g-CS to enhance solubility and optimize pharmacological effects. Ongoing comprehensive *in vivo* trials and clinical studies will further investigate these applications, aiming to provide insights and establish a foundation for the prevention and treatment of colibacillosis.

## Data Availability

The data presented in the study are deposited in the Figshare Dryad Digital Repository repository, accession number https://doi.org/10.6084/m9.figshare.27118692.v1.
